# *Lethrus* (*Lethrus*) *schneideri* sp. n. (Coleoptera, Geotrupidae) from Greece

**DOI:** 10.3897/zookeys.339.6132

**Published:** 2013-10-03

**Authors:** David Král, Oliver Hillert, Dana Drožová, Petr Šípek

**Affiliations:** 1 Charles University in Prague, Faculty of Science, Department of Zoology, Viničná 7, CZ-128 43 Praha 2, Czech Republic; 2 Kieferndamm 10, D-15566 Schöneiche bei Berlin, Germany

**Keywords:** *Lethrus*, new species, Geotrupidae, Lethrinae, Mediterranean, Palaearctic region

## Abstract

*Lethrus (Lethrus) schneideri* Král & Hillert, **sp. n.** from Thrace, Greece, is described. The new species is morphologically most similar and probably closely related to *Lethrus (Lethrus) apterus* (Laxmann, 1770) and *Lethrus (Lethrus) ares* Král, Rejsek & Schneider, 2001. Diagnostic characters (shape of mandibles, ventral mandible processes, pronotum and parameres) are illustrated. Character matrix for separation of males of the *Lethrus* species closely related to *Lethrus schneideri* Král & Hillert, **sp. n.** and geographic ranges for all species studied are mapped.

## Introduction

The genus *Lethrus* Scopoli, 1777, is a Palaearctic geotrupid genus that has a wide distribution range and occurs from central and south-eastern Europe, including the Balkan Peninsula and western part of Turkey in the west, to Mongolia and the Ninxia province of China in the south-east (cf. e.g., Král and Nikolajev 2006, Král et al. 2001, Nikolajev 2003, Král and Hillert 2013). About 120 species are currently known, all flightless and with coalescent elytra. Most of them exhibit strictly allopatric distribution ranges restricted commonly to relatively small areas (cf. e.g., Král and Hillert 2013, Nikolajev 2003). So far, investigations of this genus in the Balkan Peninsula are relatively scanty. The first three species, *Lethrus (Lethrus) elephas*, *Lethrus (Lethrus) raymondi* and *Lethrus (Lethrus) schaumii*,have been described by Reitter as late as 1890. The next species, *Lethrus (Lethrus) fallax*, has been discovered and described 85 years later by Nikolajev in 1975. Recently, based on systematic investigations by the present authors and by the Italian coleopterist Riccardo Pittino, five additional species have been recognized and described: *Lethrus (Lethrus) ares* Král, Rejsek & Schneider, 2001; *Lethrus (Lethrus) liviae* Pittino, 2011 and *Lethrus (Lethrus) halkidikiensis* Král & Hillert, 2013; *Lethrus (Lethrus) perun* Král & Hillert, 2013 and *Lethrus (Lethrus) strymonensis* Král & Hillert, 2013. *Lethrus* specimens collected from the Balkan Peninsula were also studied in parallel by standard molecular analysis methods and results obtained indicate significant differences between populations meriting for at least several of them having the “species status” (Drožová et al. in prep.).

A new and morphologically clearly different species was found in the material obtained mostly by the present authors during their expeditions to Greece between 2009 and 2012. The species was named *Lethrus (Lethrus) schneideri* and described below.

## Material and methods

The following abbreviations identify the collections housing the material examined (curators are given in parentheses).

DKCP David Král’s collection, Praha, Czech Republic, deposited in NMPC

JSCP Jan Schneider’s collection, Praha, Czech Republic

OHCB Oliver Hillert’s collection, Schöneiche bei Berlin, Germany

MNHN Muséum national d’Histoire naturelle, Paris, France (Olivier Montreuil)

NMPC National Museum, Praha, Czech Republic (Jiří Hájek)

PTCL Pavel Turek’s collection, Lanškroun, Czech Republic

ZSCK Zdeno Lucbauer’s collection, Kettering, United Kingdom

Genitalia of three males of the new species were dissected for examination. The material was examined with an Olympus SZ61 stereo microscope; measurements were taken with an ocular grid. Photographs were taken using a Canon 550D digital camera equipped with a Canon MP-E 65/2.8 MACRO lens with 5:1 optical magnification. Final images were composed from multiple partially focused images using Zerene Stacker (Zerene Systems LLC, Richland, WA, USA). Specimens of the presently described species are provided with one red printed label: “Lethrus (Lethrus) schneideri sp. nov., HOLOTYPUS, ALLOTYPUS or PARATYPUS, David Král & Oliver Hillert det. 2013”. The exact label data are cited for the material; individual lines of each label are separated by a single slash (/), [p] – preceding data within quotation marks are printed. The authors’ remarks and additional comments are enclosed in brackets.

The material was obtained mainly during the following expeditions to Greece (participants in parentheses): Greece, April 2009 (Dana Drožová, David Král, Hana Podskalská-Šípková, Petr Šípek and Aneta Venderová-Fuchsová) and Greece, April 2011 (Stephan Gottwald and Oliver Hillert).

The nomenclature used to describe morphological structures is that proposed by Pittino (2011) and Král and Hillert (2013).

## Taxonomy

### Lethrus (Lethrus) schneideri

Král & Hillert sp. n.

http://zoobank.org/C68F5659-F842-4D35-9CB9-565948451E57

http://species-id.net/wiki/Lethrus_schneideri

**Figures 1.**
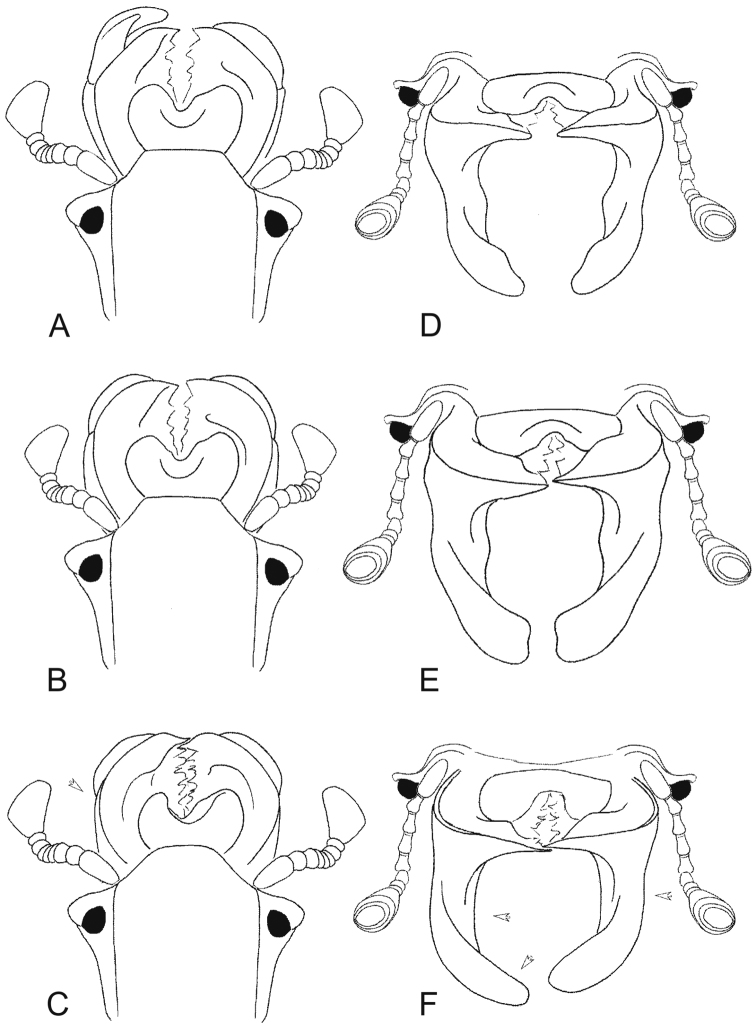
Maximally developed males: **A, D** *Lethrus (Lethrus) apterus* (Slovakia, Kamenica nad Hronom, DKCP) **B, E** *Lethrus (Lethrus) ares* (Greece, Evros dist., Polía, holotype, NMPC) **C, F** *Lethrus (Lethrus) schneideri* sp. n. (holotype). **A–C** head in dorsal aspect **D–F** head in frontal aspect. Differential characters shown by arrow. Schematically, not to scale.

**Figures 2.**
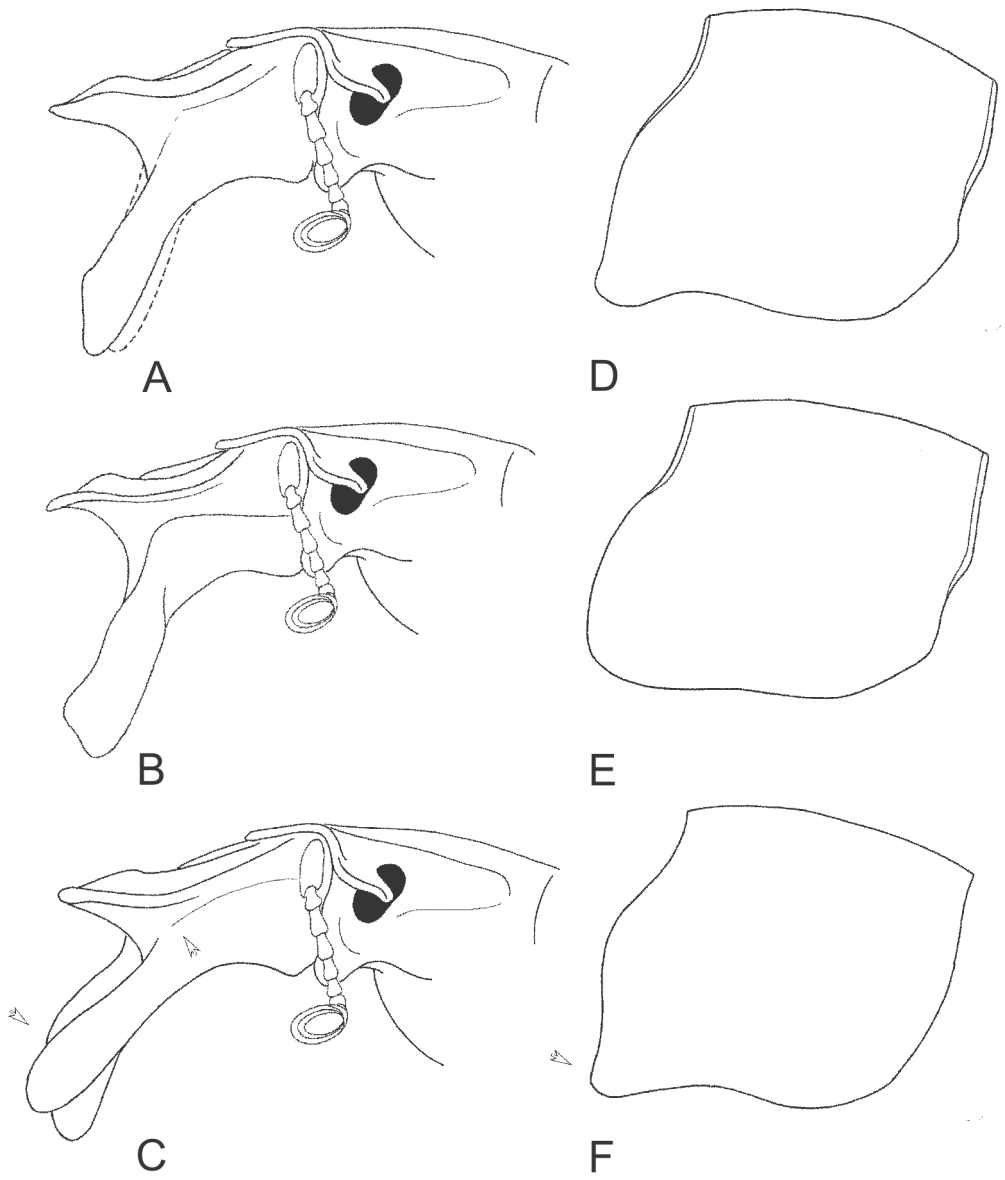
Maximally developed males: **A, D** *Lethrus (Lethrus) apterus* (Slovakia, Kamenica nad Hronom, DKCP) **B, E** *Lethrus (Lethrus) ares* (Greece, Evros dist., Polía, holotype, NMPC) **C, F** *Lethrus (Lethrus) schneideri* sp. n. (holotype). **A–C** head in left lateral aspect **D–F** pronotum in left lateral aspect. Differential characters shown by arrow. Schematically, not to scale.

**Figures 3.**
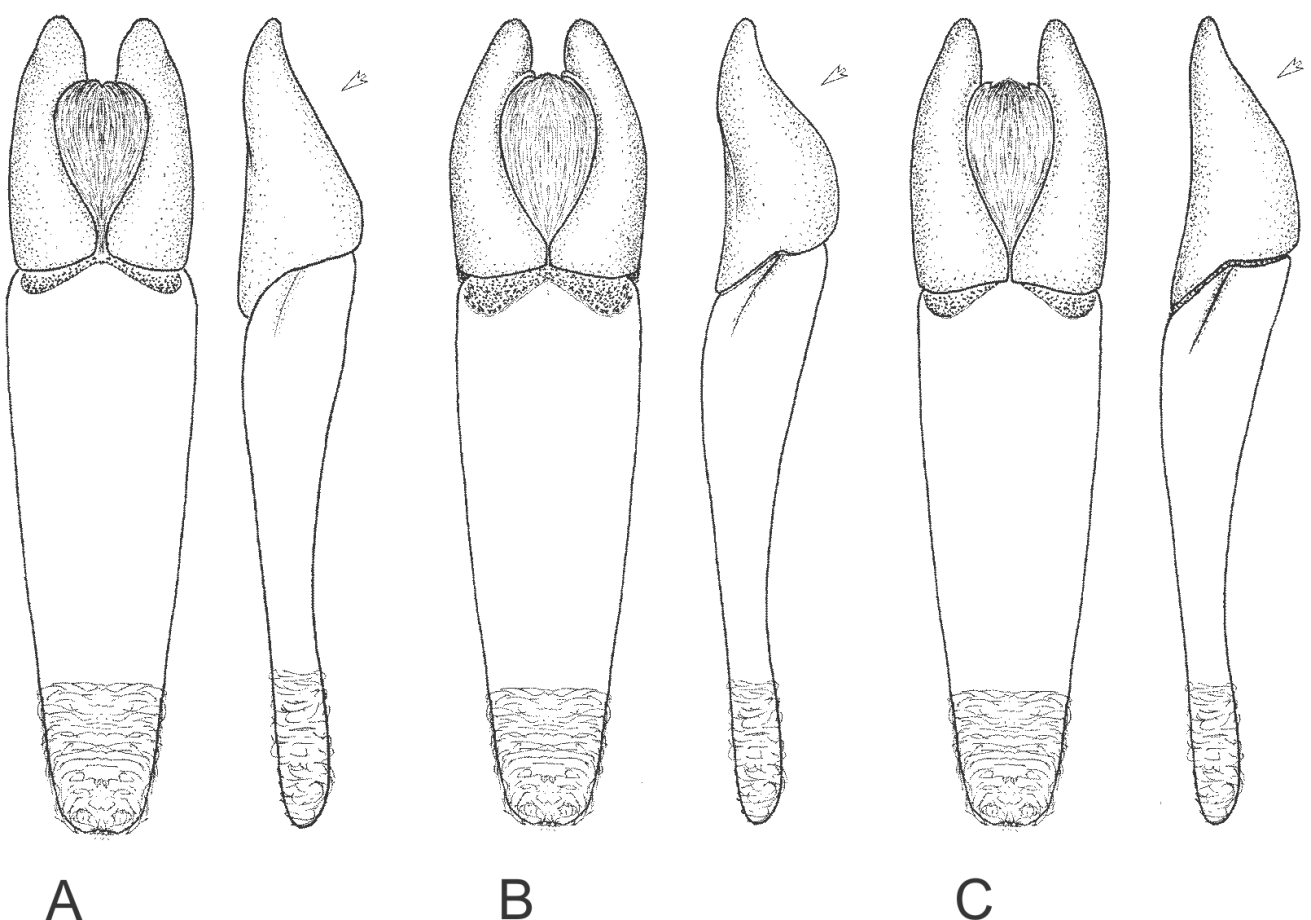
Aedeagi in dorsal and lateral aspect: **A** *Lethrus (Lethrus) apterus* (Slovakia, Kamenica nad Hronom, DKCP) **B** *Lethrus (Lethrus) ares* (Greece, Evros dist., Polía, holotype, NMPC) **C** *Lethrus (Lethrus) schneideri* sp. n. (holotype). Differential characters shown by arrow. Schematically, not to scale.

**Figures 4.**
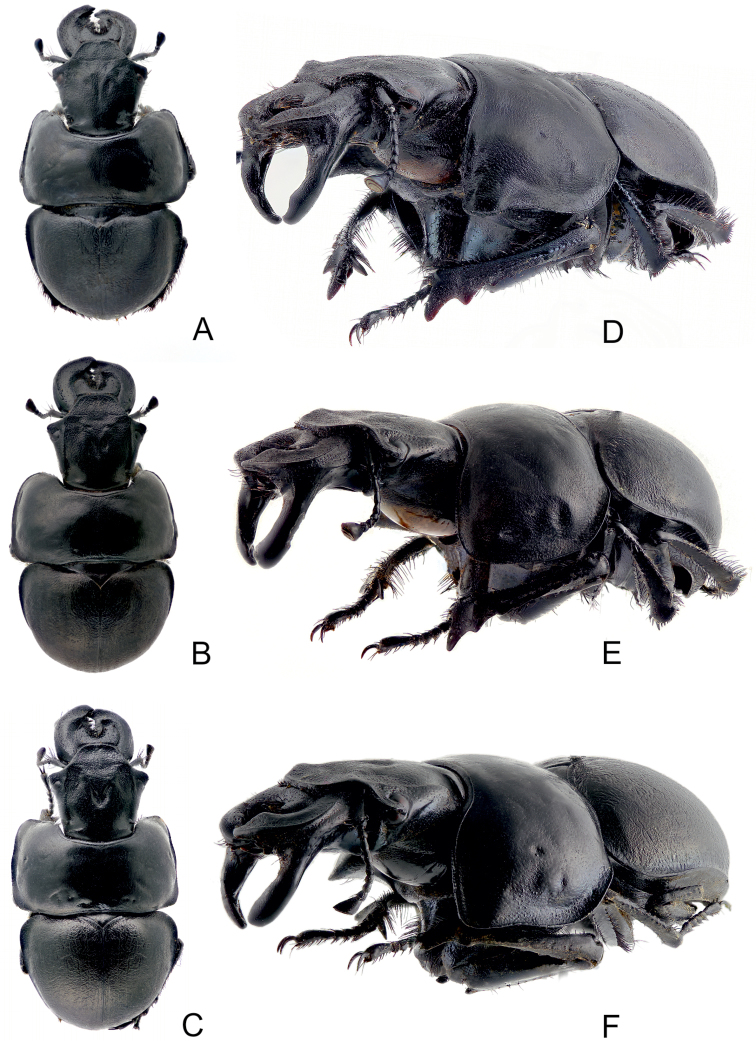
Habitus of maximally developed males: **A, D** *Lethrus (Lethrus) apterus* (Slovakia, Kamenica nad Hronom, body length: 27 mm, DKCP) **B, E** *Lethrus (Lethrus) ares* (Greece, Evros dist., Polía, body length: 28 mm, holotype, NMPC) **C, F** *Lethrus (Lethrus) schneideri* sp. n. (holotype). **A–C** dorsal aspect **D–F** left frontolateral aspect.

**Figures 5.**
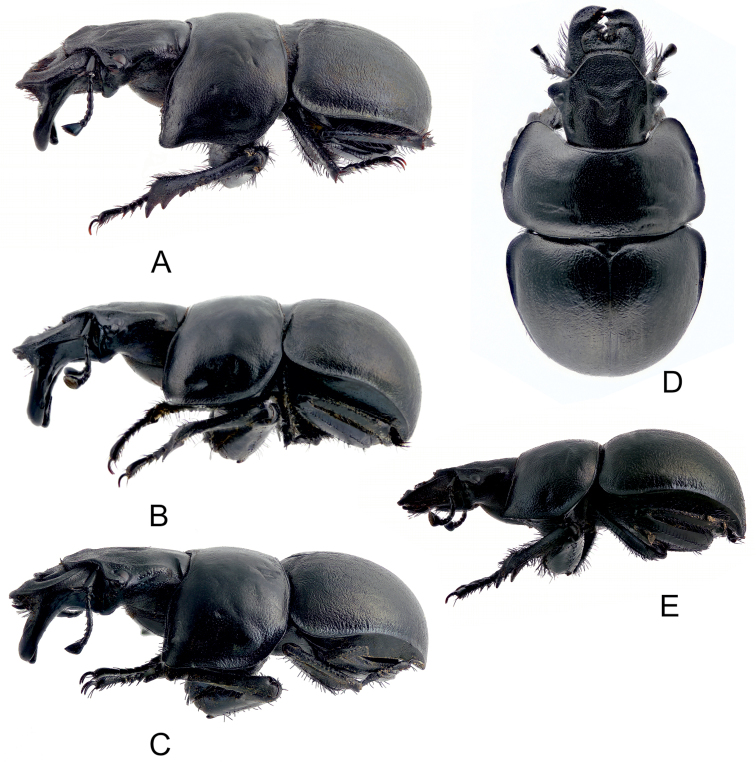
Habitus: **A** *Lethrus (Lethrus) apterus* (Slovakia, Kamenica nad Hronom, body length: 27 mm, male, DKCP) **B** *Lethrus (Lethrus) ares* (Greece, Evros dist., Polía, body length: 28 mm, male holotype, NMPC) **C** *Lethrus (Lethrus) schneideri* sp. n. (male holotype), **D–E** the same but female allotype. **A–C, E** left lateral aspect **D** dorsal aspect.

Figures 1C, F ; 2C, F ; 3C , 4C, F ; 5C–E ; 7 ; 8A–B

#### Type locality.

Greece, E Macedonia & Thrace province, Rhodope district, Komotiní environment, Karydia, approx. 120 m a.s.l., 41°06.10'N, 25°24.58'E (Fig. 8B).

#### Type material

(169 specimens). **Greece**: Holotype ♂, allotype ♀ (DKCP), “GR, E Macedonia & Thrace, 19.iv. / Rodopi dist., Komotiní env., / **KARYDIA**, 41°06.10'N, 25°24.58'E / D. Král, D. Drožová, H. Podskalská, P. Šípek & A. Venderová lgt., 2009 [p] ”. Paratypes: 5 ♂♂, 10 ♀♀ (DKCP), 3 ♂♂, 3 ♀♀ (JSCP), same data; 47 ♂♂, 20 ♀♀ (OHCB), 4 ♂♂, 2 ♀♀ (DKCP), 2 ♂♂, 2 ♀♀ (JSCP), “Greece, (Thrace), / N of Komotini, military area / 10.04.2011, leg. O. Hillert [p]”; 29 ♂♂, 10 ♀♀ (PTCL) 1 ♂, 1 ♀ (JSCP), “Greece, Thracie / Komotini, 2,1 km SZ Karydia / 41.155846, 25.422836 / 29.4.2012, leg. Pavel Turek [p]”; 14 ♂♂, 22 ♀♀ (ZLCK) 1 ♂ 1 ♀ (JSCP), “Greece, Komotini / 2,1 Km SZ Karydia / 41°8'59.19"N, 25°25'31.14"E / 29.4.2012, leg. Z. Lucbauer [p]”.

#### Additional material examined.

6 specimens). **Greece**: 1 ♂, 2 ♀♀ (NMPC), 1 ♂, 1 ♀ (OHCB), “Xanthi, Gr. / 14.v.1937 / coll. Barton [p]”; 1 ♂ (MNHN), “Grèce [p]”.

#### Description of holotype.

Maximally developed male with well developed ventral mandible processes (Figs 4C, F; 5C). Total body length 29 mm. Oblong, strongly convex; dorsal surface black, moderately shiny, except almost alutaceous pronotum; ventral surface black with fine blue tinge, moderately shiny, claws black-brown; macrosetation black.

Head (Figs 1C, F; 2C; 4C, F; 5C). Labrum bilobed, asymmetrical, right lobe remarkably more developed; surface rugosely and coarsely, shallowly and sparsely punctate, each puncture bearing short recumbent macroseta; anterior margin with dense row of long macrosetae. Clypeus transverse, trapezoidal with anterior angles round. Frontal impressions vague, frontal tubercles indistinct. Frontoclypeal suture present only laterally; keels separating eye canthus from frons only slightly developed but distinct, slightly divergent posteriad. Eye canthus exceeding eyes, projecting anterolaterad, almost rectangular, lateral margins divergent posteriad, anterolateral angle round, oblique keel above eyes absent. Pleurostomal process evenly arcuate, hardly exceeding ventrolateral mandible outline. Punctation of frons double, consisting of coarse, transversally rugose, regularly and densely distributed punctures, intermixed with fine, irregularly distributed ones; coarse punctures separated by approximately less than their diameter, punctation becoming distinctly sparser posteriad and on occiput; clypeus and eye canthus distinctly rugose.

Mandibles symmetrical, external outline almost semicircular, pointed subapically in dorsal aspect (Figs 1C, 4C) with maximum width approximately at middle of mandibles length.

Ventral mandible processes (Figs 1F, 2C, 4F, 5C) weakly asymmetrical, right process slightly more developed than left one and with different angle in lateral aspect. Both processes distinctly longer than length of mandible; base thickened, not exceeding lateral mandibular outline in dorsal aspect, with slightly concave external outline in basal half in frontal aspect; longitudinal keel on base laterally present, straight and distinctly subparallel to lateral mandibular outline, approximately as broad as maximum width of mandibles outline basally; in lateral aspect weakly arcuate, approximately subparallel to lateral mandibular outline, slightly divergent gradually basad approximately from middle of its length. Inferiobasal tooth absent; both processes bent inward approximately in middle of mandibles length in frontal view; anterior subapical tooth absent; apical emargination absent; apical tooth round.

Pronotum (Figs 2F; 4C, F; 5C) transverse, distinctly broader than base of elytra, broadest just behind middle; margin entirely bordered, slightly crenulate in anterior parts. Anterior angles weakly but distinctly projecting anterolaterad, with angulate outline; lateral margin approximately weakly emarginate anteriorly, then straight to round posterior angle; basal margin straight. Punctation of dorsal surface simple, consisting of deep, sparsely and irregularly distributed punctures; punctures separated by approximately two to four their diameters discally, surface near lateral margins considerably shagreened and alutaceous.

Scutellar shield widely triangular, finely shagreened.

Elytra almost semicircular, apices not prominent, each apex forming independent arc. Epipleuron strongly narrowed apicad, epipleural keel not reaching elytral apex. Whole surface alutaceous, finely transversally rugose; striae not indicated, entirely vanishing in rugosities.

Legs. Profemur not armed, protibia with row of eight gradually proximad diminishing external denticles, and with row of tubercles on ventromedial edge.

Aedeagus as in Fig. 3C.

#### Variability in males.

Body length 19–30 mm. Mandible processes in medium developed and underdeveloped (hypothelic) males short, more or less straight with simply rounded to almost acute apically.

#### Females

(body length 18–24 mm, allotype 24 mm – Figs 5D, E) differ from males as follows: external outline of mandibles almost straight, in apical quarter round in dorsal aspect (Fig. 5D); ventral mandibular process absent (Fig. 5E); protibia broader, row of tubercles on ventromedial edge less pronounced.

#### Differential diagnosis.

**Figure 6.**
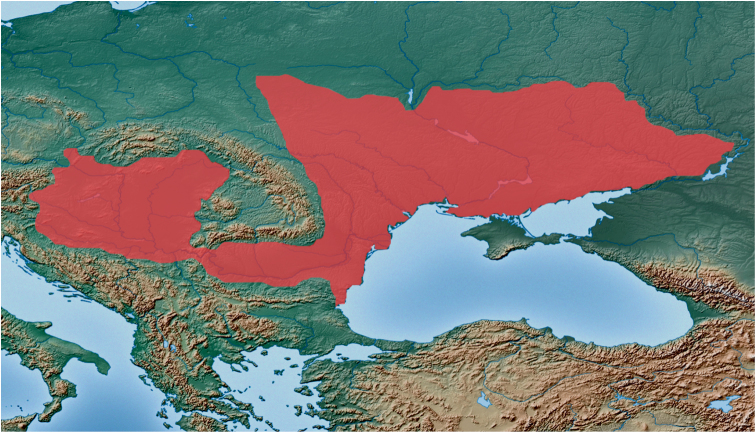
Sketch map of eastern and south-eastern part of Europe with known distribution of *Lethrus (Lethrus) apterus*. Compiled from the following sources: Baraud (1992) – overall range; Burakovski et al. (1983) – Poland; Endrődi (1957) – Carpathian basin; Guéourguiev and Bunalski (2004) – Bulgaria; Horion (1958) – Austria; Juřena et al. (2008) – Czech Republic, Slovakia; Mikšić (1970) – Serbia; Nikolajev (2003) – overall range; Panin (1957) – Romania; Semenov-Tian-Shanskij and Medvedev (1936) – overall range. Base map source: http://www.naturalearthdata.com/downloads/10m-raster-data/.

**Figure 7.**
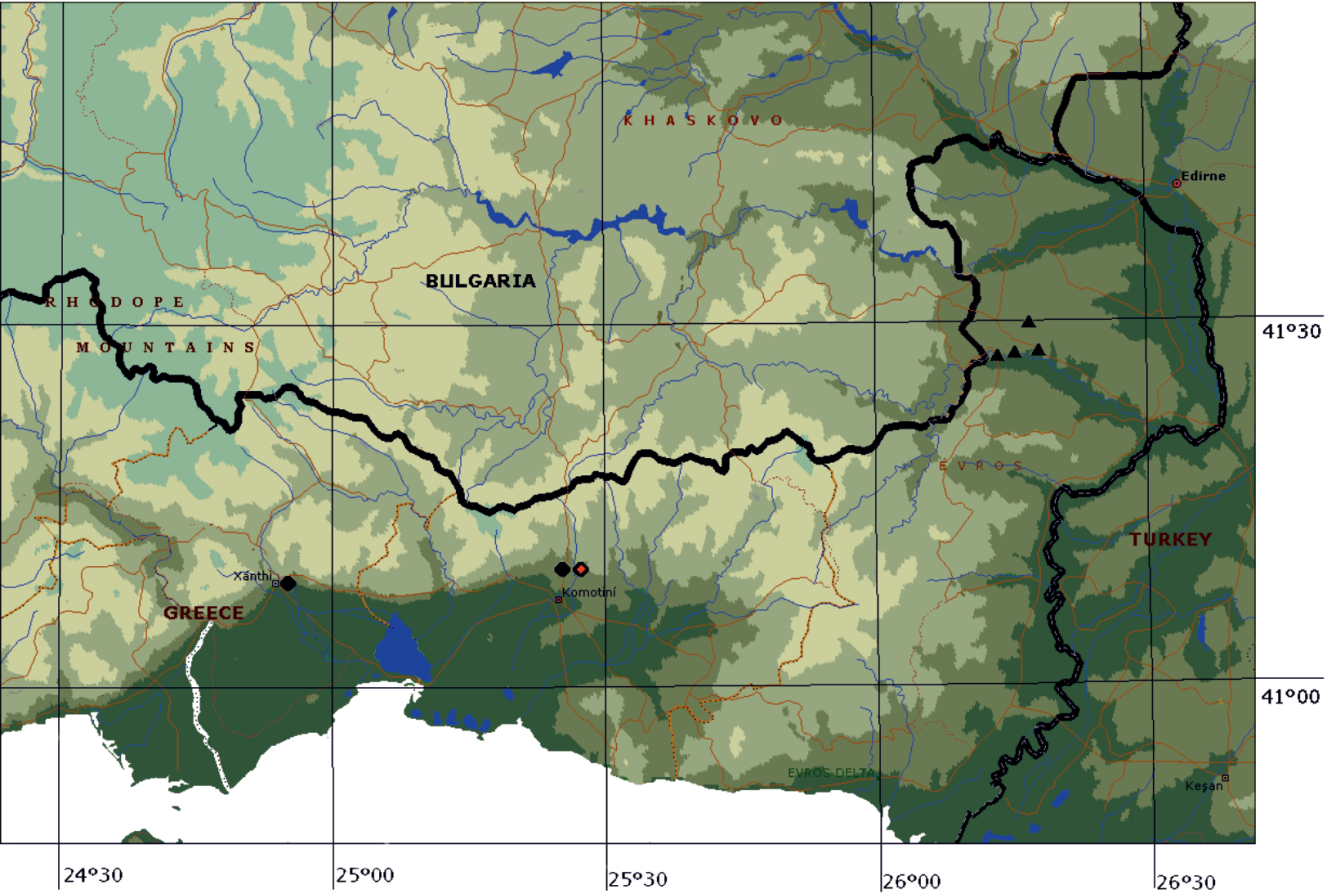
Sketch map of north-eastern part of Greece with marked distribution of *Lethrus (Lethrus) ares* – triangles, compiled from Král et al. (2001) and *Lethrus (Lethrus) schneideri* sp. n. – circles, red circle represents the type locality.

Among the species distributed in the Balkan Peninsula, the new species is most similar and probably closely related to *Lethrus (Lethrus) ares* Král, Rejsek & Schneider, 2001 and *Lethrus (Lethrus) apterus* (Laxmann, 1770). Distinguishing features are: absence of anterior subbasal tooth of ventral mandibular processes (*Lethrus (Lethrus) schaumii* Reitter, 1890 and *Lethrus (Lethrus) elephas* Reitter, 1890 have distinct anterior subbasal tooth); absence of anterior subapical tooth of ventral mandibular processes (*Lethrus (Lethrus) halkidikiensis* Hillert & Král, 2013, *Lethrus (Lethrus) perun* Hillert & Král, 2013, *Lethrus (Lethrus) raymondi* Reitter, 1890 and *Lethrus (Lethrus) strymonensis* Hillert & Král, 2013 have distinct anterior subapical tooth); presence of approximately symmetrical ventral mandibular processes and regularly round or obtuse-angular anterior pronotal angles (*Lethrus (Lethrus) fallax* Nikolajev, 1975 and *Lethrus (Lethrus) liviae* Pittino, 2011 have remarkably asymmetrical ventral mandibular processes and strongly produced acute-angular anterior pronotal angle). For characters to separate *Lethrus (Lethrus) apterus*, *Lethrus (Lethrus) ares*, and *Lethrus (Lethrus) schneideri* sp. n. see the character matrix (Table 1). Additionally, *Lethrus (Lethrus) schneideri* sp. n. is probably an endemic species of the southernmost slopes of the Rhodope Mountains approximately between the towns of Xánthi and Komotiní, while *Lethrus (Lethrus) ares* is known so far only from four spots all situated in the Eridropótamos river basin (Fig. 7) and *Lethrus (Lethrus) apterus* is a widely distributed Pannonian species known from Burgenland (Austria), Moravia (Czech Republic) and Serbia in the west to the Don river basin in the east (Fig. 6). The geographic range of the latter is separated from that of the new species by the Thracian lowlands in Bulgaria inhabited by *Lethrus (Lethrus) schaumii*,and by the Rhodope Mountains.

**Table 1.** Character matrix for separation of males of *Lethrus (Lethrus) apterus*, *Lethrus (Lethrus) ares* and *Lethrus (Lethrus) schneideri* sp. n.

**Species character**	***Lethrus (Lethrus) apterus***	***Lethrus (Lethrus) ares***	***Lethrus (Lethrus) schneideri* sp. n.**
lateral longitudinal keel on base of ventral mandible process in dorsal aspect	straight and approximately parallel to lateral mandibular outline, distinctly broader as maximum width of mandibles outline basally (Fig. 1A)	straight and distinctly subparallel to lateral mandibular outline, approximately as broad as maximum width of mandibles outline basally (Fig. 1B)	straight and distinctly subparallel to lateral mandibular outline, approximately as broad as maximum width of mandibles outline basally (Fig. 1C)
lateral longitudinal keel on base of ventral mandible process in lateral aspect	weakly arcuate, approximately parallel to lateral mandibular outline, divergent gradually basad approximately from middle of its length (Figs 2A, 4D, 5A)	almost straight, distinctly subparallel to lateral mandibular outline, distinctly divergent gradually basad approximately from middle of its length (Figs 2B, 4E, 5B)	weakly arcuate, approximately subparallel to lateral mandibular outline, slightly divergent gradually basad approximately from middle of its length (Figs 2C, 4F, 5C)
mandibular processes	both processes symmetrical (Figs 1D, 2A)	both processes symmetrical (Figs 1E, 2B)	weakly asymmetrical, right process slightly more developed than left one and with different angle in lateral aspect (Figs 1F, 2C)
shape of left ventral mandible process in lateral aspect	anterior subapical tooth present, round; apical tooth not projected apically (Figs 2A, 4D, 5A)	anterior subapical tooth present, broadened distad, angulate; apical tooth projected apically (Figs 2B, 4E, 5B)	anterior subapical tooth absent, apical tooth not projected apically (Figs 2C, 4F, 5C)
shape of ventral mandible process in frontal aspect	external outline concave basally, inferiobasal tooth present, round; subapical tooth distinct, apical emargination present, remarkably deep (Fig. 1D)	external outline strongly concave basally, inferiobasal tooth present, round; subapical tooth distinct, apical emargination present, shallow (Fig. 1E)	external outline concave basally, inferiobasal tooth absent; subapical tooth absent, apical emargination absent (Fig. 1F)
shape of anterior pronotal angle	projected anterolaterad, angulate (Figs 2D; 4A, D; 5A)	not projected anterolaterad, broadly round (Figs 2E; 4B, E; 5B)	projected anterolaterad, angulate (Figs 2F; 4C, F; 5C)
shape of parameres in lateral aspect	shallowly sinuate distally (Fig. 3A)	distinctly sinuate distally (Fig. 3B)	shallowly sinuate distally (Fig. 3C)
distribution pattern	widely distributed from Austria (Burgenland), Czech Republic (Moravia) and Serbia to approximately right bank of the Don river in the eastern Ukraine, suthernmost to the northern foothills of the Stara planina Mts in Bulgaria (Fig. 6)	restricted only to south-easternmost foothills of the Rhodope Mts (Eridropótamos river basin region in Greece) (Fig. 7)	restricted only to southernmost foothills of the Rhodope Mts (Komotiní and Xánthi regions in Greece) (Fig. 7)

#### Collecting circumstances.

**Figures 8.**
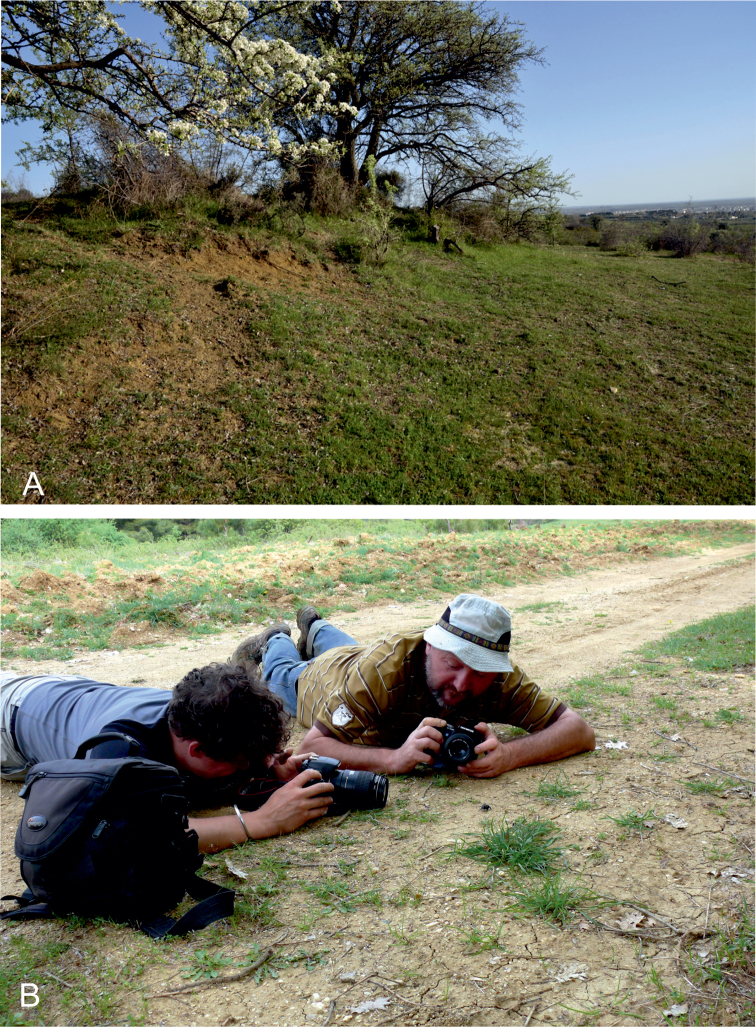
**A** Collecting habitat of *Lethrus (Lethrus) schneideri* sp. n., Greece: Thrace distr., N of Komotiní, April 2011 (photo by Oliver Hillert) **B** Type locality of *Lethrus (Lethrus) schneideri* sp. n., Greece: Thrace distr., Karydia, April 2009 (left PŠ, right DK) (photo by Hana Podskalská-Šípková).

The type series was collected from uncultivated fields on moderately steep, approximately SE oriented slope consisting of loess soil (Figs 8A–B) in a millitary area.

#### Distribution.

Greece: Thrace, southernmost foothills of the Rhodope (Ροδόπη) Mountains. (Fig. 7), the regional units of Rhodope and Xánthi.

#### Name derivation.

Patronymic, named in honour of our longtime friend, entomologist Jan Schneider (Praha, Czech Republic), an excellent Geotrupidae and Silphidae specialist.

## Supplementary Material

XML Treatment for Lethrus (Lethrus) schneideri
